# mTOR Links Environmental Signals to T Cell Fate Decisions

**DOI:** 10.3389/fimmu.2014.00686

**Published:** 2015-01-20

**Authors:** Nicole M. Chapman, Hongbo Chi

**Affiliations:** ^1^Department of Immunology, St. Jude Children’s Research Hospital, Memphis, TN, USA

**Keywords:** mTOR, T cells, iNKT cell, T_reg_ cells

## Abstract

T cell fate decisions play an integral role in maintaining the health of organisms under homeostatic and inflammatory conditions. The localized microenvironment in which developing and mature T cells reside provides signals that serve essential functions in shaping these fate decisions. These signals are derived from the immune compartment, including antigens, co-stimulation, and cytokines, and other factors, including growth factors and nutrients. The mechanistic target of rapamycin (mTOR), a vital sensor of signals within the immune microenvironment, is a central regulator of T cell biology. In this review, we discuss how various environmental cues tune mTOR activity in T cells, and summarize how mTOR integrates these signals to influence multiple aspects of T cell biology.

## Introduction

T lymphocytes are comprised of heterogeneous populations that include conventional αβ T cells, γδ T cells, invariant natural killer T (iNKT) cells, and Foxp3^+^ regulatory T (T_reg_) cells. These functionally and phenotypically distinct T cell populations are involved in immune homeostasis and tolerance, pathogen clearance, and elimination of cancerous cells. T cell fate decisions are shaped by environmental signals received from nutrients, growth factors, cytokines, and cell–cell interactions. The serine/threonine kinase, mechanistic target of rapamycin (mTOR; formerly known as the mammalian target of rapamycin), integrates these environmental cues. The mTOR kinase exists in two, multi-protein complexes: mTOR complex 1 (mTORC1) where mTOR associates with Raptor, or mTOR complex 2 (mTORC2) where Rictor and mSin1 bind mTOR (1, 2). mTORC1 activity is sensitive to, while mTORC2 activity is largely insensitive to, rapamycin treatment. Additionally, the upstream activating stimuli and downstream effector functions differ between these complexes (1, 2).

While the signaling pathways inducing mTORC2 activation in T cells are poorly understood, in other cell lineages, mTORC2 associated with ribosomes is strongly activated, while ER stress or GSK3-β-mediated phosphorylation of Rictor inhibits its activation (3, 4). Upstream positive regulators of mTORC1 activation include the PI3K–PDK1–Akt pathway, the RasGRP–Ras–MAPKK (also known as MEK)-ERK1/2 kinase cascade, and the small GTPase, RHEB. By contrast, the phosphatase, PTEN, TSC1/TSC2, and the LKB1–AMPK pathway antagonize mTORC1 function (1, 2).

When activated, mTORC1 signaling promotes S6K function and suppresses 4E-BP1 activation, while mTORC2 regulates Akt, SGK1, and PKC catalytic activity (1, 2, 5–8). mTOR signaling also activates transcription factors, such as c-MYC, hypoxia inducible factor 1-α (HIF1-α), and sterol regulatory element-binding proteins (SREBPs) (1, 2). Ultimately, the activation of mTOR-induced pathways impacts gene expression, protein translation, cell metabolism, growth, proliferation, survival, or migration in multiple cell lineages, including T lymphocytes (1, 2). Because of these critical biological effects, dysfunctional mTOR signaling is also linked to autoimmunity, obesity, and cancer, among other conditions (2, 9, 10).

Here, we review the multifactorial roles of mTOR in T cell biology. We first discuss how different environmental stimuli activate mTOR within T cells. Second, we describe the role of mTOR in thymocyte development. We then reveal how mTOR function is coupled to peripheral T cell quiescence, functional activation, and differentiation. The ability of mTOR to dampen the immune response by modulating T_reg_ cell function is also discussed. We then review the known functions mTOR serves in regulating T cell trafficking under homeostasis and upon infection. Finally, we highlight how future studies will further advance our understanding of mTOR functions in T cells, and how these findings may be applied therapeutically.

## Multiple Signals within the Immune Microenvironment Tune mTOR Activity in T Cells

**Figure 1:**
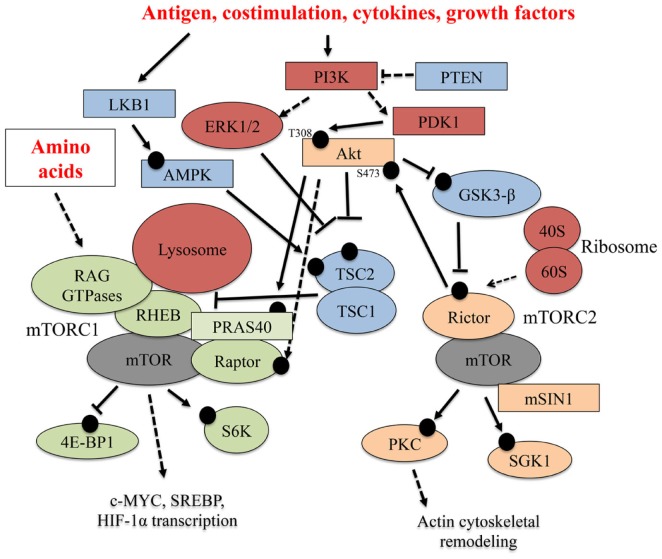
**Select upstream regulators and downstream effectors of mTOR signaling**. Multiple signaling pathways emanating from the TCR, co-stimulatory receptors, cytokines, and nutrients (amino acids) tune mTOR activation in T cells. In this figure, black circles represent phosphorylation events. Solid arrows indicate a direct, activating phosphorylation event mediated by an upstream kinase, while dashed arrows indicate an indirect, positive regulatory role for a protein in a particular pathway. Inhibitory phosphorylation events or control of pathway activation are indicated by solid or dashed flat-ended arrows, respectively.

Specialized signals derived from immune microenvironments shape T cell biology. To develop into mature T cells or gain effector functions, T cells require stimulation by immune receptors, including the TCR and co-stimulatory receptors. Soluble factors, such as cytokines, adipokines, growth factors, and nutrients, also affect T cell development and functional activation (1). mTOR integrates these immunological and environmental cues to ultimately shape T cell development, activation, and differentiation into effector or long-lived, antigen-experienced memory T cells. Below, we discuss how various factors within the immune microenvironment tune mTOR activity, and a select summary of these pathways is shown in Figure 1.

### TCR and co-stimulatory receptors

When occurring in the presence of co-stimulation, TCR recognition of self and non-self peptides expressed in the context of MHC molecules is critical for T cell development and functional activation, respectively (11–15). TCR and co-stimulatory receptor triggering activate mTOR in multiple thymocyte populations, peripheral CD4^+^ and CD8^+^ T cells, and Foxp3^+^ T_reg_ cells. Many studies have aimed to elucidate the mechanisms underlying this activation. PI3K catalyzes the conversion of membrane-bound phosphatidylinositol (PtdIns)-(4,5)-bisphosphate (PIP_2_) into PtdIns-(3,4,5)-triphosphate (PIP_3_), which acts as a second messenger to recruit the enzymes, PDK1 and Akt, to the plasma membrane (13). As we discuss in greater detail below, the PI3K–PDK1–Akt signaling axis promotes mTORC1 activation by inactivating the TSC1/TSC2 complex, thereby driving RHEB activation (2). However, downstream of the TCR, RHEB is only required for early (e.g., during the first 4 h of stimulation) mTORC1 activation (16), suggesting further mechanisms by which PI3K–Akt regulates mTORC1 activation. In this regard, PRAS40 is a steric inhibitor of mTOR, and its direct phosphorylation by Akt releases its suppressive activity and promotes mTOR activation (17). Akt also indirectly promotes mTOR phosphorylation by inducing IκB kinase α (IKKα) activity, resulting in the formation of stable Raptor–mTOR interactions that support mTORC1 function (18). The requirement for Akt in regulating mTOR activation may differ between different T cell populations, as TCR-induced mTORC1 activity is controlled by a PI3K–PDK1-dependent, Akt-independent pathway in effector CD8^+^ T cells (19). This Akt-independent pathway is linked to IL-2 induced metabolic reprograming and T cell proliferation (20). PI3K–Akt signaling is antagonized by PTEN, and loss of PTEN enhances mTOR activation (1, 2). Thus, the PI3K signaling axis is a critical regulator of mTORC1 activation at multiple levels.

In addition to PI3K, the modification of membrane-associated lipids is also controlled by PLC-γ1. Early after TCR stimulation, PLC-γ1 is activated, resulting in the cleavage of PIP_2_ into inositol triphosphate (IP_3_) and diacylglycerol (DAG). DAG supports the functional activation of the RasGRP–Ras–MAPKK–ERK1/2 pathway (13), and may cooperate with mTORC2 to induce PKC-θ activity (5). The Ras–MAPKK–ERK1/2 pathway promotes mTORC1 activation via the ERK1/2-dependent phosphorylation of TSC2 (21). T cells that lack DAG kinase (DGK)-α and DGK-ζ, which terminate DAG signaling, have elevated mTORC1 and mTORC2 activation (22). However, whether the DAG–RasGRP–Ras–MAPKK–ERK1/2 pathway acts independently or in concert with PI3K signaling is unknown, as the catalytic function of PI3K positively regulates TCR-induced ERK1/2 activation in mouse and human T cells (23, 24). Inducible Tec kinase (Itk), which directly phosphorylates and activates PLC-γ1, also promotes TCR-induced mTOR activation by inducing microRNAs that suppress PTEN expression (25). These studies indicate that many signaling pathways regulate mTOR activity downstream of the TCR.

Although TCR stimulation is necessary for effective T cell development and activation, co-stimulatory receptors must also be ligated to fully promote these processes and overcome a state of TCR-induced hypo-responsiveness called anergy (13). The classical co-stimulatory receptor for naïve T cells is CD28, which binds CD80–CD86 on antigen presenting cells (APC). However, other co-stimulatory receptors are expressed on activated T cells and T_reg_ cells, including OX40 and ICOS (26). OX40 has been demonstrated to augment TCR-induced PI3K activation to potentiate and sustain mTORC1 activity (27), further demonstrating the critical importance of the PI3K pathway in tuning mTOR activation.

Non-enzymatic proteins also regulate mTOR activation in response to antigen and co-stimulation. The CARD-containing membrane-associated protein 1 (CARMA1)-mucosa-associated lymphoid tissue lymphoma translocation protein 1 (MALT1) scaffolding complex is a recently identified, positive regulator of mTORC1 activation (28, 29). Because IKKα is known to associate with these proteins (14), this scaffolding complex may regulate the IKKα-dependent phosphorylation of mTORC1 in T cells. Additionally, the Hsp90 chaperone protein prevents Raptor protein degradation, thus promoting mTORC1 activation downstream of the TCR (30). However, the detailed mechanism by which Hsp90 prevents Raptor degradation remains unexplored.

### Cytokines

The cytokine milieu is another crucial environmental component regulating T cell fate decisions. Within the thymus and in the periphery, IL-7 signaling via IL-7R drives T cell development and homeostasis, respectively (31). In a STAT5-dependent manner, IL-7 promotes low, transient mTORC1 activation that is critical to support IL-7 function in conventional T cells (32, 33). IL-12 activates mTOR via a STAT4-dependent mechanism in activated CD8^+^ T cells (34), while IL-4 and IL-1 promote mTOR activation in T_H_2 and T_H_17 cells, respectively, to induce cell cycling (35, 36). The cytokine IL-15 regulates memory T cell formation (31, 37); however, although it activates mTOR via the PI3K pathway, IL-15-induced mTOR activation driving naïve, CD8^+^ homeostatic proliferation is not necessary for memory T cell formation (38). Finally, IL-2 is a crucial cytokine that induces clonal expansion in activated T cells and supports T_reg_ cell development and function (31). After cells express high levels of the high affinity IL-2 receptor (e.g., CD25 coupled with CD127), IL-2 signaling strongly activates transcriptional and metabolic reprograming via the Jak3–STAT5 and PI3K–Akt–mTORC1 pathways (1, 31). Itk is also required for efficient mTOR activation following IL-2 stimulation via mechanisms that are not fully elucidated (25). Like co-stimulatory receptor signaling in conventional T cells, IL-2 signaling also synergizes with TCR-dependent signals to enhance mTOR activation in T_reg_ cells (1, 39).

### Amino acids

As we will discuss throughout this review, amino acids also regulate T cell activation. Relatively little is known about how amino acids control mTOR activation in T cells, but RHEB is an essential regulator of amino acid-induced mTORC1 activation in other cell lineages (40, 41). Mechanistically, amino acids drive mTORC1 activation by recruiting the heterodimeric complex of GTP-bound RAG GEFs (RAGA, RAGB, RAGC, and RAGD) to the lysosomes via the Ragulator complex (40, 41). This process is antagonized by the GAP activity of TSC2, which, when associated with lysosomes in the absence of PI3K–Akt signaling, inactivates RHEB (42). Indeed, TSC1-deficient T cells have hyper-elevated mTORC1 signaling (43), but it should be noted that amino acids can activate mTORC1 in a TSC1-independent fashion in other cell lineages (44).

Precisely how amino acids regulate T cell responses remains uncertain. In the absence of TCR and CD28 stimulation, amino acids promote mTORC1 activation in effector CD8^+^ T cells (45). Moreover, amino acids enhance TCR and CD28-induced mTORC1 activation (29), and IL-7 or TCR and IL-2 stimulation also increases amino acids transport to promote efficient CD8^+^ T cell responses (45). However, TCR and CD28-induced mTORC1 activation is controlled by RHEB-dependent and RHEB-independent mechanisms (16). One potential explanation for these data is that amino acids localize mTORC1 to the lysosome to potentiate the early activation of mTORC1 via RHEB. After prolonged antigen exposure, however, other TCR and CD28-induced signaling pathways are sufficient to sustain mTOR activation independently of RHEB (16). Future work will continue to dissect the mechanisms by which amino acids activate mTORC1 in T cells and other cell linages, but they may regulate CARMA1–MALT1–Bcl10 complex composition and function (28, 29).

### NOTCH

NOTCH signaling promotes thymocyte proliferation and survival, and aids in their differentiation into terminally differentiated T cells (15). We discuss the process of thymocyte development in greater detail in the next section. Ligation of NOTCH activates mTOR activation through PI3K–Akt (46). Interestingly, aberrant NOTCH signaling is observed in both human and murine T cell acute lymphoblastic leukemia (T-ALL), and NOTCH inhibition in T-ALL lines suppresses mTOR activation by inhibiting c-MYC expression (47). However, the precise mechanisms by which this occurs remain undefined.

### Leptin and Sphingosine 1-phosphate (S1P)

Leptin is an adipocyte-derived cytokine, or adipokine, and serves multiple roles in T cells as discussed throughout this review. Recently, it was demonstrated that leptin receptor signaling contributes to the high levels of mTORC1 signaling that inhibits their IL-2-induced proliferation *in vitro* (39, 48). We describe how mTOR controls T_reg_ cell development, differentiation, and function in a later section. The lipid chemokine, S1P, signals via S1PR1 and drives mTORC1 activation in a PI3K–Akt-dependent manner (49–51). These studies indicate that multiple, immune-mediated signals regulate mTOR activation within T cell populations. Below, we discuss how the integration of these signals via mTOR regulates T cell development, functional activation, suppressive function, and migration.

## Role of mTOR Signaling in Thymocyte Development

### Overview of thymocyte development

T cell development occurs within the thymus and results in the generation of mature, conventional αβ CD8^+^ or CD4^+^ T cells or non-conventional T cell populations, including CD4^+^ Foxp3^+^ thymic-derived T_reg_ (tT_reg_) cells, γδ T cells, and iNKT cells. Thymocytes destined to become any T cell lineage begin as CD4^−^CD8^−^ double negative (DN) thymocytes, which can be further divided into substages: DN1, DN2a, DN2b, DN3a, DN3b, and DN4. NOTCH signals drive early proliferation and T cell lineage commitment by inducing expression of the pre-TCR (e.g., a rearranged TCRβ chain with a surrogate α chain) or the γδTCR in DN thymocytes. DN2 cells that upregulate the expression of the γδTCR in the presence of high levels of IL-7R signaling will become mature γδ T cells. By contrast, to develop into conventional αβ T cells, the DN3a cells must receive signals through the pre-TCR and NOTCH to undergo β-selection. DN cells next progress into the CD4^+^CD8^+^ double positive (DP) stage. Then, these cells receive positive and negative selection signals from the TCR to become CD4^+^ or CD8^+^ single positive (SP) cells. These SP will migrate to peripheral tissues as quiescent, mature CD4^+^ or CD8^+^ T cells. Foxp3^+^ tT_reg_ cells differentiate from DP cells upon receiving intermediate affinity TCR signals in the presence of IL-2 and/or IL-15. The coordination of receptor-mediated signals and transcription factor networks driving T cell development are discussed in other reviews (14, 15).

iNKT cells are a specialized, non-conventional subset of αβ T cells, and are harmful or protective in a variety of diseases (12). In both humans and mice, the TCR repertoire is restricted to Vα18–Jα18 chain paired with a limited number of Vβ chains (12). This TCR recognizes lipid antigens expressed in the context of the non-classical MHC molecule, CD1d. iNKT cell development also occurs in the thymus, diverging from the conventional αβ T cells at the DP stage in response to strong, CD1d-presented TCR signals in combination with SLAM ligation (12). In mice, the development of these cells is tracked by the expression of CD24, CD44, and NK1.1: immature stage 0 (CD24^+^CD44^−^NK1.1^−^), transitional stages 1 (CD24^−^CD44^−^NK1.1^−^) and 2 (CD24^−^CD44^+^NK1.1^−^), and mature stage 3 (CD24^−^CD44^+^NK1.1^+^). The transcription factors PLZF, GATA3, T-bet, and ROR-γt are expressed at different levels in these stages, determining their IL-4-producing NKT-2, IFN-γ-producing NKT-1, and IL-17-producing NKT-17 cell fate commitments (12, 52). NKT-2, NKT-17, and NKT-1 cells are enriched in stages 1/2, stage 2, and stage 3, respectively (52).

### mTOR controls conventional αβ T cell development

**Figure 2:**
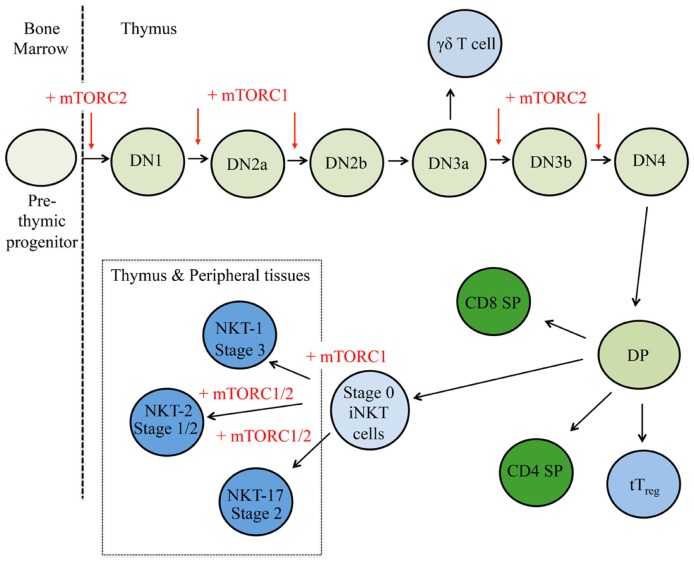
**mTOR is a critical regulator of thymocyte development**. T cell progenitors first develop within the bone marrow and migrate to the thymus. Here, cells respond to multiple environmental stimuli and progress through CD4^−^CD8^−^ double negative (DN) stages 1–4 to the double positive (DP) stage. These DP thymocytes will then adopt different cellular fates in response to additional cues. Red arrows indicate where mTORC1 and/or mTORC2 control thymocyte fate decisions, where plus signs (+) represent positive regulation and minus signs (−) depict negative regulation.

To date, many studies have determined the impacts of mTOR inhibition at different stages of thymopoiesis. The conditional deletion of Raptor early during thymocyte development results in less cell cycling and proliferation, more apoptosis, and severe thymic atrophy (53). By contrast, abrogation of mTORC1 function does not appear to affect later stages of thymocytes development, as no major developmental defects are observed when mTOR is deleted in the DP stage (54) or when Raptor is deleted in the DN3 or DP stage by Lck-Cre and CD4-Cre, respectively (16, 53). Thus, mTORC1 activation serves different functions throughout thymocyte development (Figure 2).

mTORC2 is also critical for thymocyte development, but it appears that the mechanisms by which mTORC2 supports thymocyte development differ from mTORC1 (Figure 2). Three different genetic models (e.g., whole animal, hematopoietic-specific deletion, and T cell precursor-specific deletion) have shown loss of Rictor at different stages compromises thymocyte development and leads to thymic atrophy (53, 55, 56). Mechanistically, mTORC2 activity is connected to the stability, *de novo* synthesis, and/or posttranscriptional modifications of proteins involved in thymic selection, including CD4, CD8, pre-TCR, TCR, NOTCH, and CD147, a receptor expressed on highly proliferative DN4 cells (56). Collectively, these studies reveal discrete functions of mTORC1 and mTORC2 in regulating thymocyte development.

Negative regulators of mTOR also influence T cell development. *Pten*^−/−^ T cells undergo malignant transformation regulated, in part, by elevated Akt and mTOR activation (57, 58). PTEN deficiency does not affect conventional T cell development, although only CD4 SP thymocyte frequencies were reported (59). However, another study demonstrated that loss of PTEN leads to the accumulation of DN, DP, and CD4 SP thymocytes, and a reduction in negative selection at the DP stage (60). These data are in subtle contrast to the positive roles Akt and mTOR play in thymocyte development (16, 53–55, 61, 62). Work from our lab and others have shown that T cell-specific deletion of TSC1 does not inhibit thymocyte development (43, 63, 64). By contrast, *Lkb1*^−/−^ thymocytes have a severe developmental block linked to defects in proliferation and survival (65, 66), but these effects appear to be independent of the known substrates of LKB1, AMPK1α or the related protein, MAP/microtubule affinity-regulating kinase 2 (MARK2) (65, 67, 68). Whether LKB1 controls thymocyte development via AMPK-independent pathways or AMPK family members are functionally redundant in thymocyte development is currently unresolved.

### mTOR supports non-conventional iNKT cell and T_reg_ cell development

#### γδ T cell

Treating human peripheral blood mononuclear cells with rapamycin increases the TCR-driven expansion and effector functions of γδ T cell (69), while rapamycin treatment *in vivo* suppresses the functional activation of skin-resident, murine γδ T cells (70). However, the functional role mTOR signaling serves in γδ T cell development is currently unknown.

#### iNKT cells

mTORC1 and mTORC2 are critical regulators of iNKT cell development. *Rptor*^−/−^ iNKT cells accumulate in stages 0 and 1, leading to a severe reduction of mature iNKT cells in the periphery (71, 72), whereas *Rictor*^−/−^ iNKT cells are developmentally blocked at stage 2 (73, 74). The lineage commitment of iNKT cells is compromised by loss of Raptor, as the frequency of IFN-γ-producing, T-bet^+^ NKT-1 cells is reduced (72). By contrast, Rictor deficiency does not diminish NKT-1 cell differentiation. Loss of Rictor, however, does suppress NKT-17 cell and/or NKT-2 cell development (73, 74). Mechanistically, mTORC1 regulates iNKT cell proliferation (72), whereas mTORC2 drives TCR-induced proliferation at stage 1 and protects from TCR-induced apoptosis (73, 74). These data indicate that mTORC1 and mTORC2 serve important, yet distinct, functions in iNKT cell development.

Elevated mTOR signaling also alters iNKT cell development. Compared to conventional T cells, iNKT cells express higher levels of *Tsc1* and *Tsc2* mRNA (75). Importantly, this high level of TSC1/TSC2 expression regulates the terminal maturation of iNKT cells, as *Tsc1*^−/−^ thymocytes have severe limitations in developing past stage 2 and into functional NKT-1 cells (75). Recent work has also demonstrated that folliculin-interacting protein 1 (Fnip1) is required for iNKT cell progression beyond stage 2 (76). Mechanistically, *Fnip1*^−/−^ iNKT cells are more sensitive to apoptosis, which may be attributed to excessive mTOR signaling and mitochondrial disruption (76). Finally, PTEN also regulates iNKT cell development and function. Suzuki and co-workers demonstrated that PTEN deficiency blocks progression from stage 2 to stage 3 and also abrogates TCR-induced IFN-γ production in these cells (77). Moreover, we have recently demonstrated that NKT-17 cell development is enhanced in the absence of PTEN, in part because mTORC2 signaling is elevated in these cells (74). These studies demonstrate a pivotal role for mTOR signaling in controlling iNKT cell development.

#### Foxp3^+^ tT_reg_ cells

In addition to iNKT cells, Foxp3^+^ tT_reg_ represent a non-conventional T cell population that develops within the thymus (14). It has been reported that mTOR conditional knockout mice have normal frequencies of T_reg_ cells (54). Conditionally deleting PTEN within T cells does not dramatically alter T_reg_ cell development, although PTEN does suppress the IL-2-induced expansion of these cells (59). Moreover, TSC1 deficiency within the total T cell or T_reg_ cell compartments does not alter thymic or peripheral T_reg_ cell ratios (43, 78), but does impair their function as we discuss below. It is noteworthy that these studies did not distinguish between tT_reg_ and peripherally induced T_reg_ cells (pT_reg_), which differentiate from naïve CD4^+^ T cells following antigen stimulation in the presence of select cytokines. We discuss the pharmacological and genetic evidence linking mTOR signaling to pT_reg_ differentiation later in this review. Additional studies should explore the effects of LKB1–AMPK signaling on tT_reg_ cell development.

## mTOR Controls Peripheral T Cell Homeostasis, Activation, and Differentiation

**Figure 3:**
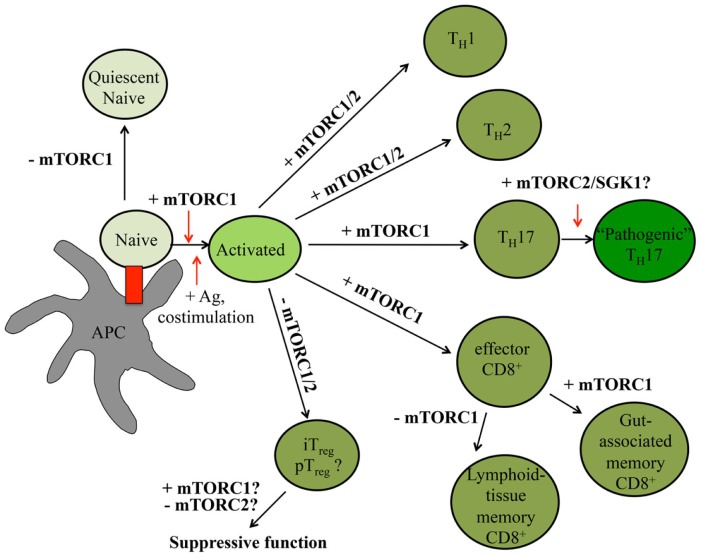
**mTOR signaling controls peripheral T cell fate decisions**. In the peripheral tissues, T cell quiescence is controlled by low levels of mTORC1 signaling. Upon receiving antigen and co-stimulatory signals, T cells rapidly expand. In the presence of select cytokines, CD4^+^ T cells further differentiate into different effector CD4^+^ T cell lineages. CD8^+^ T cells will become effector T cells before becoming memory T cells. The roles mTORC1 and mTORC2 serve in various T cell states are indicated within the figure, with positive roles shown with plus (+) signs and negative roles indicated by minus (−) signs. Question marks (?) indicate pathways requiring further investigation.

In the periphery, naïve T cells undergoing IL-7–IL-7R-driven homeostatic proliferation are maintained in a quiescent state (11). Upon receiving the appropriate antigen, co-stimulatory, cytokine, and nutrient signals, these T cells rapidly proliferate, generating multiple, antigen-specific T cell clones capable of inducing effective adaptive immune responses (13, 79, 80). These signals also induce the expression of transcription factors, including T-bet, GATA3, ROR-γt, Bcl-6, and Foxp3, which promote CD4^+^ T helper (T_H_)1, T_H_2, T_H_17, T follicular helper (T_FH_), and pT_reg_ cell differentiation, respectively (79). Similarly, these signals drive CD8^+^ T cell differentiation into short-lived effector T cells [SLECs; T-bet^hi^Eomesodermin (EOMES)^+^Blimp-1^hi^KLRG1^+^IL-7Rα^lo^] or memory precursor cells (MPECs; T-bet^lo^EOMES^lo^Blimp-1^hi^KLRG1^lo^IL-7Rα^hi^) (80, 81). The switch from naïve to activated to memory T cells is coordinated by an intricate network of epigenetic, transcriptional, and metabolic programs, many of which are directly influenced by mTOR activation (1, 82, 83). Below, we discuss how alterations in mTOR signaling affect mature T cell quiescence, functional activation, and differentiation. A summary is shown in Figure 3.

### T cell homeostasis requires low levels of mTORC1 signaling

Tonic TCR signaling induced by host-derived antigens in combination with IL-7R signaling maintains T cell homeostasis (11). Moreover, recent work has linked PI3K–Akt–mTOR signaling to the homeostatic proliferation of NKT-17 cells, which preferentially require IL-7 for their homeostasis (84). While mTOR, Raptor, or Rictor-deficient T cells have no alterations in steady-state peripheral T cell homeostasis (16, 54), low levels of mTOR signaling appear to maintain CD4^+^ and CD8^+^ T cell quiescence. In support of this idea, *Tsc1*^−/−^ T cells have excessive mTORC1 signaling, which promotes aberrant cell cycling (43, 63, 64, 85). *Tsc1*^−/−^ T cells have reduced homeostatic proliferation in response to IL-7 signaling and are hyper-responsive to TCR-induced apoptotic signals (43, 63, 64, 85). Bcl2 overexpression rescues this defect in apoptosis, but does not restore quiescence (43).

PTEN and LKB1 are also regulators of peripheral T cell homeostasis. Mature PTEN-deficient T cells are hyper-proliferative, resistant to apoptosis, and drive autoimmunity (86). Similar to *Tsc1*^−/−^ T cells, peripheral *Lkb1*^−/−^ T cells are hyper-activated and are more sensitive to TCR-induced apoptosis (87). Moreover, anti-CD3 and anti-CD28 antibody, but not IL-7, induced proliferation is impaired in the absence of LKB1 (66). Although TSC1 and LKB1 have similar defects, multiple metabolic pathways, including mitochondrial functions, are dysregulated in *Tsc1*^−/−^ T cells (43, 63), while glycolysis is enhanced in the absence of LKB1 (87). Thus, TSC1 and LKB1 are both critical to maintain quiescence, but they control naïve T cell homeostasis by different mechanisms.

### mTOR signaling is coupled to T cell clonal expansion

It has been demonstrated that mTOR, RHEB, and Raptor-deficient T cells have defects in antigen-driven proliferation (16, 54). This effect is largely dependent upon mTORC1-mediated signaling driving cell cycle entry from quiescence, as loss of Raptor or rapamycin treatment in naïve, but not proliferating, T cells blocks clonal expansion and instead promotes T cell anergy (16, 88). Rapamycin-treated, human T cells also have reduced proliferation (89), further supporting the idea that mTORC1 is a critical regulator of T cell proliferation. Raptor-deficient T cells have reduced c-MYC and SREBP expression and activation, respectively, leading to decreased glycolysis, oxidative phosphorylation, and/or lipogenesis (16, 90).

In addition to TCR and co-stimulatory signals, amino acids also regulate mTORC1 activation to promote T cell proliferation. Depletion of select amino acids, including arginine, leucine, or tryptophan, impairs T cell proliferation (91–93). Similarly, glutamine uptake is required for efficient T cell responses, and deletion of leucine transporters, including CD98, Sla7a5, and ASCT2, reduces mTOR activation and T cell clonal expansion (29, 94–98). Interestingly, leucine and glucose import appear to be linked, as ASCT2-deficient T cells have reduced expression of the glucose receptor, Glut1 (29). This observation may functionally link amino acid sensing to proliferation, as glucose uptake and glycolysis are intimately linked to this process (99). Collectively, these studies reveal that mTOR signaling is a crucial determinant of T cell activation.

### mTOR regulates transcriptional and metabolic programs to control T cell differentiation

#### CD4^+^ T cells

In addition to driving T cell proliferation, mTORC1 and mTORC2 also serve different roles in priming effector CD4^+^ T cell differentiation in response to antigen, co-stimulatory, and cytokine signals. In the absence of mTOR function, T_H_1, T_H_2, and T_H_17 polarization are all impaired (16, 54, 89, 100–102). mTORC1 activity controls T_H_1 and T_H_17 differentiation (100, 101). However, whether mTORC2 is also required for T_H_1 generation remains controversial (100, 101). T_H_2 polarization and function are severely impaired in the absence of Raptor (16), but are retained in RHEB-deficient T cells that exhibit a partial loss of mTORC1 activity (16, 101). Interestingly, although other studies link Rictor–mTORC2 to T_H_2 differentiation, rapamycin treatment of *Rictor*^−/−^ T cells diminishes T_H_2 polarization more profoundly than Rictor deficiency alone (16, 100, 101). These data highlight the central role of mTORC1 in shaping T_H_2 differentiation.

Additional work has aimed to determine the mechanisms by which mTOR links immunological signaling to effector CD4^+^ T cell differentiation. Rapamycin treatment impairs TCR and CD28-induced T-bet and GATA3 upregulation, and also abrogates permissive de-methylation of the *Ifng* and *Il4* gene loci (103). These results may explain why deleting various mTOR-related proteins inhibits T_H_1 and T_H_2 differentiation. We have demonstrated that *Rptor*^−/−^ CD4^+^ T cells have profound defects in metabolic reprograming driven by the transcription factors, c-MYC and SREBP (16), which impairs the functional activation and differentiation of these cells. T_H_17 differentiation is reduced in the absence of HIF-1α, a transcription factor functionally regulated by mTORC1 activity (104, 105). Interestingly, although *Rictor*^−/−^ CD4^+^ T cells do not exhibit defective T_H_17 differentiation, recent studies link the mTORC2 substrate, SGK1, to the IL-23-driven generation of highly inflammatory, “pathogenic” T_H_17 cells that can promote autoimmune disease development in mice (106, 107). Future work will investigate if mTORC2 regulates IL-23R signaling to facilitate this process.

Environmental cues also signal to mTOR, supporting the differentiation of CD4^+^ T cells. It has been demonstrated that *Asct2*^−/−^ T cells have reduced T_H_1 and T_H_17 differentiation and function as a result of reduced leucine import (29, 98). This defect is linked to attenuated TCR and CD28-induced mTORC1 activation (29). Slc7a5-deficient T cells, which have impaired amino acid transport, also have reductions in T_H_1 and T_H_17 differentiation (45). Moreover, S1PR1 signaling promotes T_H_1 differentiation (51), while leptin receptor signaling drives T_H_1 and T_H_17 differentiation (108, 109). Future work will explore the detailed mechanisms by which these and other environmental signals, including additional amino acids, influence effector CD4^+^ T cell differentiation. We describe studies implicating how mTOR signaling shapes pT_reg_ differentiation in a later section of this review.

#### CD8^+^ T cells

In CD8^+^ T cells, mTORC1 inhibition or deletion increases memory CD8^+^ T cell formation or maintenance by regulating the expression of various transcription factors, including FoxO1, T-bet, and Blimp-1 (38, 110–113). Memory CD8^+^ T cells may arise due to asymmetric cell division or impaired differentiation from effector CD8^+^ T cells (81, 114). However, knocking down Raptor in activated CD8^+^ T cells also potentiates memory functional CD8^+^ T cell differentiation (113), and deleting TSC1 from activated CD8^+^ T cells impairs memory differentiation and function (115). Thus, mTORC1-mediated control of memory CD8^+^ T cell differentiation appears to be linked to defective effector to memory differentiation. mTORC1 signaling regulates CD8^+^ T cell differentiation, in part, by controlling glycolytic and oxidative phosphorylation metabolism following IL-15 stimulation (115). However, it should be noted that IL-15-independent functions for mTOR in controlling CD8^+^ T cell memory formation have been described (38). For instance, mTORC1 imparts control over effector versus memory T cell fate decisions by regulating the expression of NOTCH on naïve CD8^+^ T cells (116). Thus, mTORC1 utilizes multiple mechanisms to influence effector versus memory CD8^+^ T cell differentiation and function.

Recent data revealed a site-specific role for mTOR signaling in the generation of CD8^+^ T cell memory. Marzo and colleagues found that rapamycin treatment enhances memory CD8^+^ T cell differentiation in the blood and spleen, but the number of memory CD8^+^ T cells in the lungs and peripheral lymph nodes are not affected (117). In fact, mucosal CD8^+^ T cells isolated from the small intestine lamina propria are reduced in numbers upon rapamycin treatment, in part due to defects in T cell trafficking as discussed below. Collectively, these data indicate a critical role for mTOR in modulating tissue-specific, effector versus memory fate decisions in CD8^+^ T cells.

In response to chronic infections, CD8^+^ T cells become functionally impaired or exhausted (118). Kaech and colleagues recently demonstrated that Akt and mTOR signaling are impaired in CD8^+^ effector T cells following a chronic viral infection as compared to an acute infection (119). This event leads to the FoxO1-dependent upregulation of PD-1 and promotes the survival of terminally differentiated, exhausted CD8^+^ T cells. Signaling downstream of PD-1 antagonizes mTOR activation (120), which drives CD8^+^ T cell exhaustion (119). Consistent with this idea, PD-1 blockade restores function in exhausted, CD8^+^ T cells in an mTOR-dependent manner (119). Therefore, in addition to supporting CD8^+^ T cell effector versus memory formation, the mTOR–FoxO1 axis also regulates CD8^+^ T cell exhaustion.

## mTOR Maintains Immune Tolerance by Controlling T_reg_ Cell Function and Stability

Foxp3^+^ T_reg_ cells maintain T cell homeostasis in the periphery, and their loss of function causes severe, multi-organ autoimmunity in humans and mice (121). Interestingly, mTOR signaling serves discrete functions in T_reg_ cell differentiation and function. Several groups demonstrated that T_reg_ cell differentiation is potentiated *in vitro* (called iT_reg_ cells) in the presence of rapamycin (54, 122–128). An inhibitory role for mTOR in the generation of iT_reg_ cells was further supported using *Mtor*^−/−^ T cells (54), with mTORC1 and mTORC2 serving functionally redundant roles in suppressing iT_reg_ differentiation (54, 101). Likewise, HIF-1α deficiency enhances T_reg_ cell differentiation (104, 105). However, the functional capacities of *Mtor*^−/−^ or rapamycin-expanded T_reg_ cells require further investigation, as the source of the T_reg_ cells used in the *in vitro* suppression assays were not a highly purified population of Foxp3^+^ T_reg_ cells. The *in vivo* suppressive activity of these cells also remains largely unexplored, although rapamycin-expanded, human T_reg_ cells are functional in a xenograft transfer model (129).

Regulatory T cells have high, basal levels of mTOR signaling compared to their naïve T cell counterparts (39, 48). However, the proper threshold of mTOR signaling is critical to support their suppressive function *in vitro* and *in vivo*. We recently demonstrated that Raptor-deficient T_reg_ cells lose suppressive activity *in vitro* and *in vivo*, the latter of which contributes to rampant autoimmunity and lethality in mice (39). Mechanistically, Raptor-mTORC1 signaling is linked to cholesterol biosynthesis and lipid metabolism, processes that are important to support the expression of the T_reg_ cell effector molecules, ICOS and cytotoxic T lymphocyte antigen (CTLA)-4. These effects are not observed in T_reg_ cells lacking Rictor, and combined loss of Raptor and Rictor partially restores the suppressive function of T_reg_ cells *in vitro* and *in vivo* (39). Thus, loss of mTORC1, but not mTORC2, activity is linked to T_reg_ cell dysfunction.

While these studies show that loss of mTORC1 activity is deleterious to T_reg_ cell function, excessive mTOR signaling within T_reg_ cells also compromises their function and affects their stability. TSC1-deficient T_reg_ cells are impaired in their ability to suppress inflammatory responses, as they lose Foxp3 expression and acquire T_H_17 cell effector-like functions *in vitro* and *in vivo* (78). Consistent with this study, recent work demonstrated that patients with autoimmune diseases have elevated mTOR activation within their T_reg_ cells (10). Although they proliferate more robustly following IL-2 stimulation, *Pten*^−/−^ T_reg_ cells appear to retain their suppressive capacity *in vitro* and can suppress colitis development *in vivo* (59). However, the role of PTEN in T_reg_ cells has not been specifically addressed using a conditional deletion model. Thus, distinct negative regulators of mTOR activity appear to serve different functions in T_reg_ cells.

Several pathways have mechanistically been shown to modulate mTOR activity within T_reg_ cells to regulate their proliferation, differentiation, and function. Leptin receptor signaling restrains TCR and/or IL-2 stimulation-induced T_reg_ proliferation *in vitro* (48, 130), suggesting that leptin levels may be a critical factor influencing T_reg_ cell proliferation *in vivo* (131). Maintenance of Foxp3 expression is required for T_reg_ suppressive function (132). Transient TCR stimulation drives PI3K–Akt–mTOR signaling that antagonizes Foxp3 expression (133), and rapamycin treatment enhances Foxp3 expression by modulating DNA methylation within the *Foxp3* locus (103). Through multiple mechanisms, T_reg_ cells can modulate amino acid availability within a microenvironment (92, 121, 134). Interestingly, mTOR inhibition and amino acid deprivation synergize with TGF-β signaling to augment Foxp3 expression *in vitro* (91, 92). Finally, S1PR1 signaling to mTORC1 restrains T_reg_ differentiation in the thymus and periphery, and limits their suppressive function *in vitro* and *in vivo* during homeostasis and inflammation (50, 51).

## mTOR Regulates T Cell Trafficking

**Figure 4:**
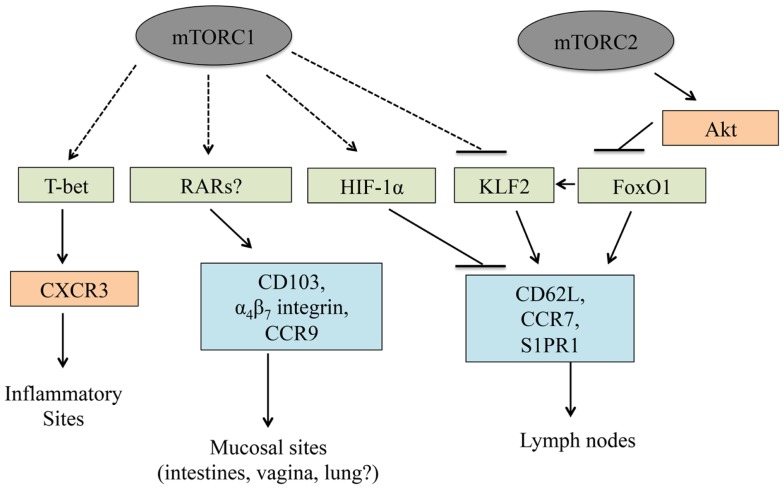
**T cell trafficking is linked to mTOR**. mTORC1 and mTORC2 control T cell trafficking by regulating the expression and/or functional activation of multiple transcription factors. In this manner, mTOR signaling regulates trafficking into inflammatory sites, lymph nodes, and mucosal sites.

After an infection occurs, chemokine and adhesion receptors localize T cells to the proper anatomical location. The adhesion receptor CD62L and chemokine receptors, CCR7 and S1PR1, allow T cells to enter and be retained in peripheral lymph nodes such that T cell activation may occur (49, 135). As with T cell development and activation, mTOR signaling is also a critical regulator of T cell trafficking. PI3K or mTORC1 inhibition in activated CD8^+^ T cells reduces IL-2-induced downregulation of CCR7, CD62L, and S1PR1 expression (136), which causes these cells to traffic to lymph nodes (34). By contrast, the downregulation of these molecules occurs more efficiently in the absence of PTEN or TSC1 (43, 115, 136, 137). These trafficking defects may partially account for why rapamycin treatment enhances and TSC1 deficiency suppresses memory CD8^+^ T cell differentiation (34, 115, 136). Although the precise mechanisms by which mTOR signaling regulates trafficking are not known, mTOR modulates the expression of Kruppel-like factor 2 (KLF2) and HIF-1α, two transcription factors that modulate the expression of lymph node homing receptors (19, 136). Further, mTORC2 may inhibit FoxO1 function by enhancing Akt activity, and FoxO1 transcriptional activity modulates the expression of lymph node homing receptors (137). Finally, mTORC1 activity induces T-bet expression (34), which drives CXCR3 upregulation and subsequently localizes T cells to sites of infection (138, 139). Thus, mTOR activity regulates T cell trafficking via multiple mechanisms (Figure 4).

Recent work also demonstrates a role for mTOR in T cell trafficking to non-lymphoid tissues. Trafficking into the gut-associated mucosa is regulated by CCR9, the α_4_β_7_ integrin, and CD103 (140). In CD8^+^ T cells, rapamycin treatment suppresses the expression and/or function of these molecules, leading to a severe reduction in these cells within mucosal sites (117). Similarly, knocking down mTOR within activated CD8^+^ T cells also reduces trafficking to the small intestine. Although it was not mechanistically determined how mTOR controls mucosal site homing, the retinoic acid receptors (RARs) induce CCR9 and α_4_β_7_ integrin expression in activated T cells (141, 142). As T_reg_ cells, T_H_17 cells, and iNKT cells play pivotal roles in gut-associated lymphoid reactions (143, 144), future work will need to explore how mTOR inhibition or hyper-activation influences trafficking to mucosal sites within these cell lineages.

## Concluding Remarks

Current work has highlighted the critical role the environmental sensor mTOR plays in T cell biology. mTORC1 and mTORC2 both support thymocyte development, but integrate distinct and overlapping signals and impart discrete functions to facilitate this process. In contrast to thymocytes, mTORC1 is the dominant regulator of the functional activation and differentiation of conventional T cells in the periphery. mTORC1 activation is critical for clonal expansion, effector CD4^+^ T cell differentiation, and T_reg_ cell function, while mTORC2 also contributes to these processes but with limited effects. However, further work is needed to determine the role mTORC1 and mTORC2 serve in the induction of site-specific immune responses, including the generation of T_FH_ cells and tissue-specific T_reg_ cell populations, the latter of which play critical functions in dampening immune responses in mucosal sites, adipose tissues, and tumors (121, 145).

From a clinical perspective, it will be critical to determine the impacts of mTOR inhibition on the specific immunity to pathogens, tumors, and auto-antigens. Hyper- or hypo-activation of mTOR has a profound impact on T cell development and activation, so these investigations will provide insight into how rapamycin, its rapalogs, and other next generation mTOR inhibitors will influence localized and systemic immune responses in different disease settings. Given the intricate link between mTOR function and T cell fate decisions, it is feasible that one could modulate mTOR activation within specific inflammatory sites and/or immune cell types to modulate the immune response in states where both mTOR and T cells are dysfunctional. These studies will be key toward determining if mTOR suppression in T cells is a viable target for treating autoimmunity, cancers, and infectious diseases, or for boosting memory CD8^+^ T cell responses to enhance vaccine efficacy.

## Conflict of Interest Statement

The authors declare that the research was conducted in the absence of any commercial or financial relationships that could be construed as a potential conflict of interest.
